# *Intellicount*: High-Throughput Quantification of Fluorescent Synaptic Protein Puncta by Machine Learning

**DOI:** 10.1523/ENEURO.0219-17.2017

**Published:** 2017-12-06

**Authors:** J. A. Fantuzzo, V. R. Mirabella, A. H. Hamod, R. P. Hart, J. D. Zahn, Z. P. Pang

**Affiliations:** 1 Child Health Institute of New Jersey, New Brunswick, NJ 08901; 2 Department of Cell Biology and Neuroscience, Rutgers University, Piscataway, NJ 08854; 3 Department of Neuroscience and Cell Biology, Rutgers University, Piscataway, NJ 08854; 4 Department of Pediatrics, Robert Wood Johnson Medical School, Rutgers University, New Brunswick, NJ 08903; 5 Department of Biomedical Engineering, Rutgers University, Piscataway, NJ 08854

**Keywords:** automated image analysis, high-throughput, immunofluorescence, machine learning, synapse formation, synapse quantification

## Abstract

Synapse formation analyses can be performed by imaging and quantifying fluorescent signals of synaptic markers. Traditionally, these analyses are done using simple or multiple thresholding and segmentation approaches or by labor-intensive manual analysis by a human observer. Here, we describe *Intellicount*, a high-throughput, fully-automated synapse quantification program which applies a novel machine learning (ML)-based image processing algorithm to systematically improve region of interest (ROI) identification over simple thresholding techniques. Through processing large datasets from both human and mouse neurons, we demonstrate that this approach allows image processing to proceed independently of carefully set thresholds, thus reducing the need for human intervention. As a result, this method can efficiently and accurately process large image datasets with minimal interaction by the experimenter, making it less prone to bias and less liable to human error. Furthermore, *Intellicount* is integrated into an intuitive graphical user interface (GUI) that provides a set of valuable features, including automated and multifunctional figure generation, routine statistical analyses, and the ability to run full datasets through nested folders, greatly expediting the data analysis process.

## Significance Statement

Synapses are functional information processing and computational units in the brain, and disordered synapse formation is associated with the pathophysiology of neurodevelopmental and neuropsychiatric disorders. Therefore, accurate measurement of the numbers and qualitative characteristics of synapses is widely employed in neuroscience and of great importance. Existing approaches commonly use immunocytochemistry for synaptic protein markers. Image analysis and quantification are often time consuming, requiring substantial user interaction with semiautomated image processing programs, which are prone to unintended user biases and processing errors. Here, we provide a novel machine learning (ML)-based image processing paradigm and software program that is fully automated and capable of high-throughput analysis for quantifying synapse numbers under varied culture types and conditions.

## Introduction

The mammalian brain is composed of billions of neurons connected to each other by trillions of synapses, which govern information flow in the brain and thus control cognition and brain-related behaviors. Aberrant synapse formation has been implicated in a wide range of neurodevelopmental and neuropsychiatric disorders, including autism spectrum disorders, schizophrenia, and many others (Sekar et al., 2016; Südhof, 2008; Tsai et al., 2012). Morphologically, the synaptic connections composed of both presynaptic and postsynaptic components under light microscopy appear as puncta-like structures. Considerable effort has been invested in developing techniques and platforms to accurately identify and quantify synapse numbers and other characteristics of synaptic proteins to unravel the molecular mechanisms by which synapses form and function under normal and abnormal conditions. Although some of these techniques can be very specialized, analysis of synaptic puncta by imaging of synaptic proteins by immunofluorescent (IF) labeling is among the most established and commonly used technique because it is informative, and relatively easy to perform. This type of analysis offers insight into the number, distribution within subcellular compartments (pre- or postsynaptic, dendritic spines, shafts, and somatic), and other characteristics of synaptic protein complexes. The synapse density (number of synapses per unit length or area of dendrite), intensities of the synaptic protein IF signals, and the sizes of synaptic puncta often correlate with functional synaptic parameters as measured by electrophysiology (Ko et al., 2009; Lee et al., 2013; Zhang et al., 2015; Chen et al., 2017). Indeed, the size and intensity of the excitatory postsynaptic density protein 95 (PSD-95) puncta tend to correlate with measures of synaptic strength (El-Husseini et al., 2000). A handful of useful tools have been developed to facilitate image-based analysis of protein puncta associated with neural structures (Schätzle et al., 2012; Danielson and Lee, 2014).

However, several major limitations of existing tools available for analyzing synaptic protein puncta include the accuracy and reliability as well as the ease of use for performing such analyses, particularly when large datasets are involved. Commonly used methodologies often involve manual or semiautomated object tracing and region of interest (ROI) measurements to identify relative differences between experimental conditions. Experimenter-assisted manual quantification methods (Ippolito and Eroglu, 2010) are often cumbersome and require tedious repeated work. While this approach can be accurate, it can be subject to human error and bias and depends on the skill of the experimenters. Therefore, several commercial (e.g., MetaMorph, Molecular Devices) and open-source semiautomated software programs (e.g., ImageJ, with different customized modules) have been developed. These platforms generally employ thresholding and segmentation paradigms to identify puncta and neural processes (Schätzle et al., 2012). Recent work has extended semiautomated processing techniques to include multiple thresholds (Danielson and Lee, 2014), which significantly improves separation of adjacent IF puncta clusters. However, for puncta whose full area is occupied by a broad grayscale range, simple thresholding may alter the size, shape and intensity because the thresholds required to separate adjacent puncta can eliminate the low-intensity pixels, which may actually demarcate the true synapse boundary. Specifically, use of higher thresholds, if left nonoptimized by the user, can artificially underestimate puncta size since the threshold required to separate closely adjacent puncta tend only to capture the highest intensity pixels of an ROI and vice versa. Additionally, most existing platforms are considered semiautomated, because they require interaction by a user with the program at multiple processing and preprocessing steps (often with each image) and do not allow automated batch processing of multiple nested folders (i.e., folders organized by condition within a single directory) or directories with images collected from different conditions. Moreover, most programs are normally not integrated with statistical capabilities. Therefore, analysis of synaptic protein puncta is often tedious and prone to experimenter bias.

To address these limitations, we developed a novel machine learning (ML)-based image processing paradigm and software program, *Intellicount*, which dramatically reduces the time required for analysis, improves processing artifacts generated by simple image thresholding, and allows image processing to proceed without the need for carefully set thresholds. We have applied this in different experimental conditions. Additionally, *Intellicount* is packaged into an intuitive graphical user interface (GUI) that is equipped with commonly used statistical analysis and graphical figure representation, further increasing the efficiency by which quantitative analyses can be performed and data can be represented. As such, we provide the research community with an open-source, easy-to-use tool for quantification of fluorescent synaptic protein puncta. This open-source platform aims to improve the reproducibility and reliability in performing such analyses.

## Materials and Methods

### IF sample preparation and image collection

Both mouse and human induced neurons (iNs) at different culture densities were used in this study. Specifically, low/medium density human iN cultures (∼100,000 neuronal cells/78.5 mm^2^) were prepared from male H1 embryonic stem cells (NIH registry WA01) as described previously for excitatory iNs (Zhang et al., 2013). Inhibitory iN subtypes were derived from C12-induced pluripotent stem cell (iPSC) lines as described previously (Yang et al., 2017). C12 is an iPSC line originally created from a male subject from the COGEND collection (Saccone et al., 2007; Bierut et al., 2008) of nicotine abuse as described previously (Oni et al., 2016). It carries a minor allele variant in the OPRM1 gene (N40D), unrelated to the current study.

Coverslips were fixed at 6 weeks *in vitro* for IF experiments. Work with human embryonic stem cells was approved by the Rutgers Embryonic Stem Cell Research Oversight (ESCRO) committee. Mouse hippocampal neurons at relatively high density (∼200,000 neurons/78.5 mm^2^) were isolated from postnatal day 0–1 C57/BL/6 background male newborn pups as described previously (Maximov et al., 2007; Chanda et al., 2017) and fixed at the different time points after culturing (4, 6, 8, 10, 12, and 16 d *in vitro*, DIV). Fixation was performed with 4% paraformaldehyde diluted in PBS for 10 min at room temperature, washed well in PBS, blocked, and permeabilized for 30 min in PBS containing 4% bovine serum albumin, 1% normal goat serum, and 0.2% Triton X-100. Coverslips were then incubated with rabbit anti-synapsin (1:3000, E028, a gift from the Südhof lab), rabbit anti-vesicular GABA transporter (vGAT; 1:500, Millipore Ab5062P, RRID:AB_2301998), and mouse anti-microtubule-associated protein 2 (MAP2; 1:500, Sigma M1406, RRID: AB_477171 and 1:1000 Millipore AB5543, RRID: AB_571049) primary antibodies for 1 h, washed well, and incubated in appropriate secondary antibodies (1:500, Alexa Fluor 546-conjugated anti-rabbit and 488-conjugated anti-mouse, Invitrogen). All steps were conducted at room temperature. Coverslips were then mounted onto slides using Fluoroshield media containing DAPI (Sigma). Approximately 101.6 × 101.6 μm^2^ Z-stack images (1024 × 1024 pixel resolution, 8-bit grayscale depth for human and mouse cultures) were acquired at 1× digital zoom using a 63× water immersion objective by laser-scanning confocal microscopy (Zeiss LSM-700, Carl Zeiss). Mouse culture images for synaptogenesis time course were taken with 16-bit grayscale depth. All images were acquired using identical laser intensity, digital gain, and offset background within each dataset. Maximum intensity projection images were then constructed and images were exported in TIFF format for analysis in *Intellicount* or Fiji, which is a distinct package of ImageJ (Schindelin et al., 2012). Subfields (∼20 × 20 μm^2^) were cropped from iN images using Fiji for analysis by manual tracing to identify puncta ROIs. Fiji-fixed thresholding was performed using threshold settings 55 for lower threshold and 255 for upper threshold. For both manual and fixed thresholding methods within Fiji, ROI properties were analyzed using Analyze Particles.

Full, uncropped images from mouse cultures were used for analysis of synaptogenesis time course. For synaptogenesis time course, a total of 29–33 images were obtained from randomly sampled fields distributed evenly over two coverslips each obtained from two independent cultures. For high density versus low neuronal density comparisons, images were taken from the same batches of hippocampal cultures at DIV 8. Fields with high cell density (more than five cell bodies) were selected for high density, and fields with low cell density (one to two cell bodies) for low density images. For the calmodulin (CaM) knock-down experiment, images were obtained from Pang et al. (2010), which were acquired at dimensions of 71.3 × 71.3 μm^2^ taken at 652 × 652 pixel resolution.

### Image processing

*Intellicount* was developed using MATLAB 64-bit R2017a. The ML process uses a looped algorithm that optimizes traces against gradient images. Segmentation is performed in most cases on a filtered image (unless the user opts to forego the filter due to high background). Images are converted to a format with a normalized intensity range from 0 to 1 for all processing. Puncta IF intensities are rescaled to a 0–255 (8-bit images) range for display. Three thresholds (analogous to 8-bit grayscale values of 30, 70, and 220) are then applied to the filtered image to generate three binary images. Next, the two upper threshold binary images are dilated according to the ML-defined dilation size. Following dilation, a “background removal factor” is applied to reduce the capture of background signals from any threshold level. This serves to remove ROIs that do not have a significantly high gradient level. We hypothesized that true signals would have a gradient value greater than the average background signal. Therefore, any ROI that did not have an average gradient intensity higher than the background removal factor (on a 0.0–1.0 intensity scale), was removed from the binary image. Increasing this factor creates a stricter environment for puncta identification, and is recommended for high background images.

After dilation, the binary images are processed through a watershed transform to separate closely located puncta. Next, if selected, the MAP2 signal segmentation and correlation is performed. MAP2 segmentation is guided by Otsu’s method (Otsu, 1979), which determines optimal threshold values based on histogram variance of grayscale pixel intensities. An additional dilation of 1.5 μm (or other distance specified by user) is added to the width of segmented dendrites to capture puncta located near dendrites. Somata are also segmented from the MAP2 signal and either included or excluded from the correlation (as specified by the user). Remaining puncta undergo size discrimination.

While synaptic structures range in size from ∼200 nm to 500 nm (Ribrault et al., 2011) as identified by electron microscopy (Siksou et al., 2007), protein puncta visualized by confocal microscopy generally range from 0.4–4 μm in diameter, based in part on the diffraction limit of light and inability to optically resolve closely adjacent terminals or spines. We established area cutoffs of 0.16 and 6.25 μm^2^ consistent with ranges used in a previous protocol (Mitchell et al., 2012). This upper size limit can be altered within the GUI.

**Figure 1.**
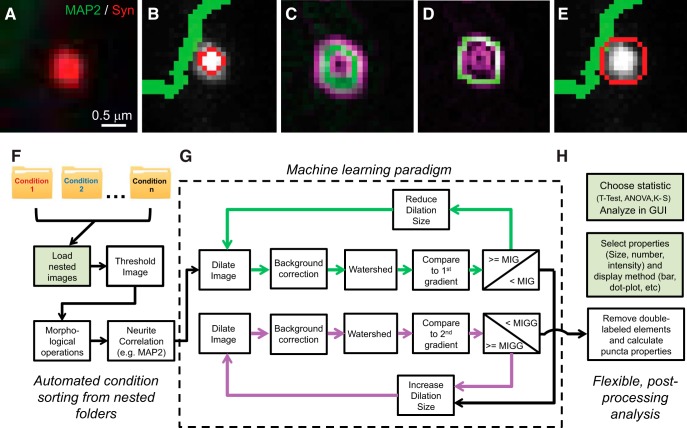
ML facilitates high-throughput image puncta analysis. ***A***, Example synaptic puncta (synapsin in red) juxtaposed to a dendrite (MAP2 in green) and ***B***, a trace provided by thresholding. ***C***, First and second gradient (green and purple, respectively) provide a template. ***D***, Trace boundary is improved by structure-guided ML (an optimized trace in green) overlaid on the second gradient (purple). ***E***, The improved puncta trace as a result of ML. ***F***, Nested folder input for image pre-processing sorted by data round (i.e., condition). ***G***, Structure-guided ML algorithm runs multiple dilation iterations to optimize traces (green and pink loops indicate processing against the first and second gradients, respectively). ***H***, Puncta properties are extracted and stored in matrix form, allowing flexible post-processing representation and analysis by the user (refer to Fig. 6 for statistical output and data display). Note, this overall paradigm allows user to interact with the program at fixed beginning and end steps (highlighted in green shaded boxes).

Since high intensity, nonoptimized thresholds tend to capture only the brightest pixels of an ROI (Fig. 1*A*,*B*), we applied our ML algorithm to improve ROI traces by comparing the overlap of all puncta traces of the image with the gradient images. Gradient images reflect the magnitude of the gradient of the original image using the imgradient command with the Sobel operator, which is a convolution mask applied to the image as described elsewhere (Sobel, 1990). Two gradient images are obtained: the first gradient which results in a single circular peak for each puncta (Fig. 1*C*, green pixels), and the second gradient which results in two circular traces (Fig. 1*C*, purple pixels). In the first round of ML, each successive ML loop alters the dilation size until there is maximum overlap between the ROI trace and the first gradient. The mean intensity value of the overlap with the first gradient is called the mean intensity gradient (MIG):

$$MIG=\frac{1}{n}\frac{1}{m}\sum_{i=1}^{n}\sum_{j=1}^{m}\nabla {(I}_{i,j})\cdot {SI}_{E,i,j}$$

where I_i,j_ is a single pixel from the original grayscale image of the puncta channel and SI_E,I,j_ is a pixel from the edge-image of the segmented puncta (i.e., the puncta trace). The mean is determined using the mean of overlap for all pixels. Similarly, the second derivative (MIGG) is

$$MIGG=\frac{1}{n}\frac{1}{m}\sum_{i=1}^{n}\sum_{j=1}^{m}\nabla^{2}{(I}_{i,j})\cdot {SI}_{E,i,j}$$

Once the highest MIG value is found, the algorithm now increases the dilation size until the mean of the puncta trace multiplied by the outermost second gradient is maximized (Fig. 1*D*). The trace that produces this maximized MIGG is used for all subsequent analysis, and the program considers the traces now optimized (Fig. 1*E*). This process begins with threshold 2 and is repeated for threshold 3.

To ensure puncta identified by multiple thresholds are not duplicated in the final analysis, all multi-labeled elements produced from the multiple thresholds (puncta labeled by more than one threshold) are then removed by identifying objects underneath the highest threshold that identified the object. The results are then flattened into one binary image. From here, the MATLAB command regionprops was used to find properties of all identified puncta. The entire process is performed automatically, allowing the user to upload image folders into the program, and after selecting the “start” button, performing plotting and statistical analyses (Fig. 1*F–H*). The user may upload a single folder of images, or a folder containing subfolders of images, which we refer to as “nested folders.” This expedites the analysis process by reducing user time uploading new batches of images for processing.

### Computational run time analysis

This analysis was conducted on 10 cropped images. To collect run times for *Intellicount*, MATLAB functions tic and toc where used. For Fiji-fixed threshold, a macro within Fiji was recorded and run over the 10 images. The getTime command was used to capture the start and end times. The Fiji-manual approach was conducted using a manual step to optimize threshold selection. The threshold selection was timed separately outside of the macro for each of the 10 images. The identified thresholds were then added to the macro code, and the macro was run. The computational time and the threshold selection time were summed to capture total time for the Fiji-manual approach.

### Statistical analysis

Data are presented as mean ± SEM and statistical analyses were performed using one-way ANOVA with Tukey-Kramer’s *post hoc* testing. Student’s *t* tests in Figure 4 were performed in Excel using two-tailed equal variance. vGAT parameters expressed in text as mean ± SEM.

### Code accessibility

The code/software described in this paper is freely available online at http://license.rutgers.edu/technologies/2018-013_intellicount. The code is available as Extended Data 1.

10.1523/ENEURO.0219-17.2017.ed1 Extended Data 1 File contains two MATLAB files and user guide. Download Extended Data 1, ZIP file.

## Results

*Intellicount* was primarily designed to allow IF image processing for quantification of synaptic puncta to proceed without the need for an experimenter to carefully set thresholds of IF signals and preselect image subfields for analysis. Building off a previous approach using multiple thresholds (Danielson and Lee, 2014), *Intellicount* uses a combination of multiple thresholding and puncta trace optimization aided by structure-guided ML. Multiple thresholds facilitate the separation of closely-located IF puncta and the inclusion of a greater range of intensities over variable background levels. Higher thresholds tend to capture the brightest regions (usually the center) of an IF punctum, leaving the less intense areas unaccounted for (Fig. 1*B*). Using the gradients as a structure guide (Fig. 1*C*), we optimized puncta traces against the gradient (Fig. 1*D*) to improve ROI identification (Fig. 1*E*). Furthermore, *Intellicount* allows users to import full image fields arranged in folders according to experimental condition or different timings (Fig. 1*F*). It then performs image pre-processing to correlate to neural structures (dendrites) before ML (Fig. 1*G*) and subsequently quantitatively analyzes the data and extracts the data for further statistical analyses with built-in statistics (Fig. 1*H*). This highly-efficient approach greatly reduces the number of human interactions and thus lesser (or no) human bias and errors would be introduced by a user (Fig. 1*F*, *H*, user interaction points shown as green shaded boxes).

### *Intellicount* improves ROI tracing and quickly quantifies puncta number and properties

**Figure 2.**
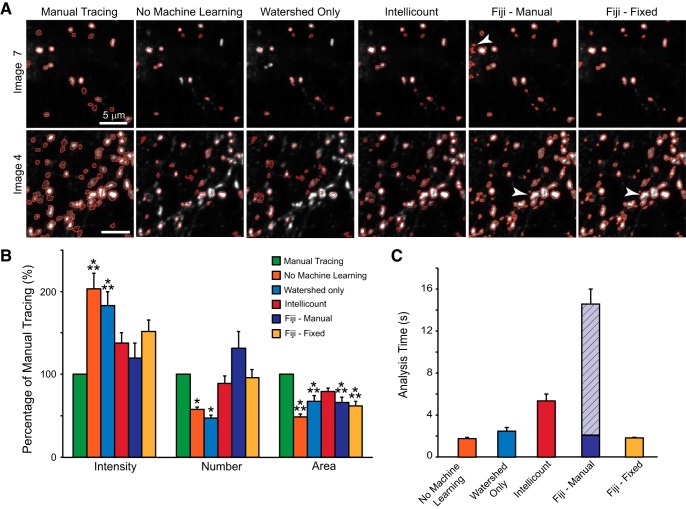
Comparison of *Intellicount*’s ML with manual traces and with Fiji. ***A***, Representative traces (red) provided by manual tracing by a human observer, three processing conditions of *Intellicount,* and user adjusted variable- or fixed-threshold analysis in Fiji. Representative low (image 7) and high (image 4) puncta density fields are shown. White arrows indicate background included as ROI (top row) and merged puncta (bottom row). Note, “no machine learning” and “watershed only” provide traces obtained in *Intellicount* using default thresholds (without and with selection of a watershed algorithm, respectively) and without application of the ML. ***B***, Comparison of these processing techniques normalized to values obtained by manual tracing. Results are displayed as mean ± SEM from analysis of 10 random fields; *p < 0.05, ***p < 0.001 using ANOVA with *post hoc* Tukey-Kramer, as compared to manual trace. ***C***, Total analysis time per cropped image performed on 10 fields. Computational analysis time is shown in solid bars and was recorded either from MATLAB (for No Machine Learning, Watershed Only, and *Intellicount*) or Fiji macro. Diagonally-lined section of bar in Fiji-manual condition highlights the fraction of analysis time used for manual threshold selection.

To test the accuracy of the synaptic structure identifications (tracing of the puncta) based on IF signals of defined synaptic proteins, we compared the synaptic IF puncta properties (synapsin and MAP2 IF images provided by *Intellicount* against manual, human traces, and single thresholding methods in Fiji). Synapsin is a well-defined presynaptic marker and was used to quantify synaptic numbers in many recent publications (Pak et al., 2015; Patzke et al., 2016; Yi et al., 2016). Manual tracing for ROI identification was performed using ImageJ and Fiji (Schindelin et al., 2012). First, we blindly identified all discernable IF puncta under high magnification, which enabled us to trace the ROI with accuracy down to the single-pixel level. *Intellicount* was then run under three different conditions: no ML, i.e., simple thresholding using its default three thresholds; watershed only, which applies a watershed algorithm to thresholded images; and the *Intellicount* default (ML condition), which applies structure-guided ML with a watershed algorithm to the thresholded images. We also compared traces obtained using two different Fiji approaches: manually-selected thresholds for individual fields and a single, manually-fixed threshold (55 lower, 255 upper), with the Analyze Particles tool within Fiji. We compared four parameters: number, area, and IF intensity, as well as average analysis time per image, over all six tracing approaches (Fig. 2*A*). Data were expressed as ratios compared to the manual trace. As expected, the ML version of *Intellicount* outperforms non-ML and watershed versions of the program on three parameters for IF puncta (Fig. 2*B*). As compared to Fiji ROI segmentation, *Intellicount* performs comparably. Our program, however, largely circumvents common problems with single thresholds even when thresholds are carefully selected, namely, inclusion of background pixels and merging of closely located puncta (Fig. 2*A*, second row, white arrows). Multiple thresholds with watershed improved the separation of closely located synaptic puncta, while single thresholds can merge puncta together, which are then evaluated as a single, large ROI. *Intellicount* improves on this, but does not fully separate all puncta as compared to manual tracing. While the areas quantified by Fiji and *Intellicount* were different from the manual trace, they were not significantly different from each other, although *Intellicount* may offer slight improvement over Fiji approaches.

In addition to defining the accuracy of *Intellicount*, we also compared computational processing time per image between methods performed in Fiji and *Intellicount* with the same set of 10 IF images. To evaluate one currently available method (single threshold segmentation using Fiji) against *Intellicount*, we used a macro within Fiji to quantify the computational run time. While runs of *Intellicount* with ML take slightly longer compared to runs without ML and to the Fiji macro, the timescale is within the same range of the other approaches, and provides the user with a greater amount of information in the process, such as data graphs (Fig. 2*C*). To show the impact of manual operations on run time, we also added a thresholding selection step to the Fiji macro run time. The addition of this manual step, while only on scale of 10–20 s (hatched bar), significantly lengthens the time for processing images. This demonstrates that user interaction can slow data analysis time and that fully automated methods for large datasets are preferable. Furthermore, when considering full image analysis with MAP2 correlation, the differences between automated and manual approaches will likely be further extended, since automated approaches, and specifically *Intellicount*, have been designed specifically for this type of analysis and performs it automatically without optimizing thresholding other image channels or the need to crop subfields from full images. Therefore, *Intellicount* performs puncta IF analysis automatically while offering both accuracy and efficiency, with immediate data readout.

### *Intellicount* identifies puncta over a wide range densities and intrinsic characteristics

**Figure 3.**
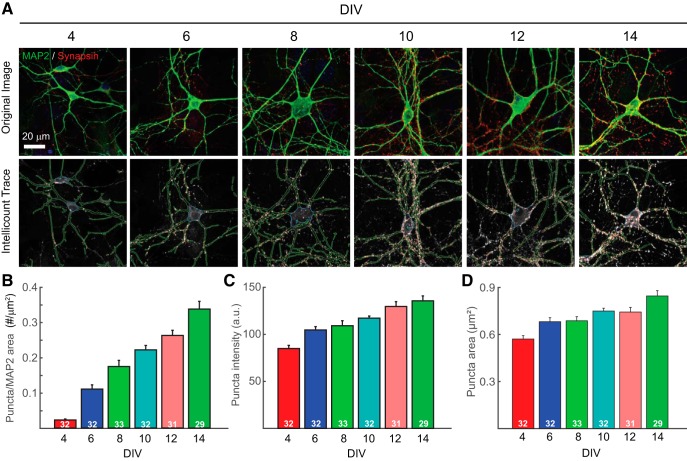
*Intellicount* recapitulates analysis of synaptogenesis over time in cultured hippocampal neurons. ***A***, Upper panels: representative images taken at increasing numbers of days in culture. Lower panels, corresponding traces provided by *Intellicount* (red, correlated puncta synapsin traces; green, segmented dendrite traces in MAP2 channel; blue, trace of soma segmented from MAP2 channel). Display of the MAP2 channel was deselected in the lower panels facilitate visualization of puncta. A total of 29–33 images were obtained from randomly sampled fields distributed evenly over two coverslips each obtained from two independent cultures. ***B–D***, Quantification and graphical representation of (***B***) synapse number, (***C***) mean fluorescence intensity, and (***D***) area provided by *Intellicount* with minor adjustments in Adobe Illustrator (renaming of “rounds” to appropriate conditions and thickening of stroke width for representation of graph and bar lines). Colors displayed are default in the program. Data are displayed as mean ± SEM. Numbers within bars indicate the number of images processed from that time point.

To demonstrate the utility of the program to handle large datasets and its ability to discern puncta covering a wide range of sizes, intensities and densities, we performed a time course analysis of synaptogenesis and synaptic maturation using primary mouse hippocampal neurons. The total dataset consists of 189 images generated from two different batches of cultures collected after increasing time spent in culture (4, 6, 8, 10, 12, and 14 DIV) with IF for synapsin (presynaptic marker) and MAP2 (dendritic marker), organized into six nested folders (each containing 29–33 images). Note that the number of the images in each nested folder can be increased dramatically as long as images could be acquired with the same parameters and format. The program ran these images with a MAP2 + Soma correlation, which includes puncta located within a defined distance from the MAP2 IF signal-positive dendrite, as well as the segmented soma. The processing was completed in ∼2.5 h using an iMac with a 2.5-GHz processor. The background removal factor was increased from the default 0.175 to 0.25 accommodate higher backgrounds found in later time points due to higher signal intensity. For output image traces, *Intellicount* displays the puncta channel in grayscale and the MAP2 channel at 50% brightness in grayscale (which can be toggled off; Fig. 3), with the trace color matching the original color of the puncta channel. MAP2 traces are also shown in their original channel color, however the soma sizes are shown using a third unused channel color. Here, synapsin IF signal was shown in red (with red program traces in lower panel), and MAP2 IF signal in green (with green program traces in lower panel; Fig. 3*A*). Somata, which do not have a distinct channel, were traced in the unused RGB color, blue. An increase in synaptic density (number of puncta per unit MAP2 area; Fig. 3*B*), as well as increases in synapse area (Fig. 3*C*) and synapsin fluorescence intensity as a function of time spent in culture were observed (Fig. 3*D*). These results are consistent with previously published data on synapse maturation in a neuronal culturing system (Mozhayeva et al., 2002; Chanda et al., 2017).

### *Intellicount* can be used under varied culture conditions and antibodies

**Figure 4.**
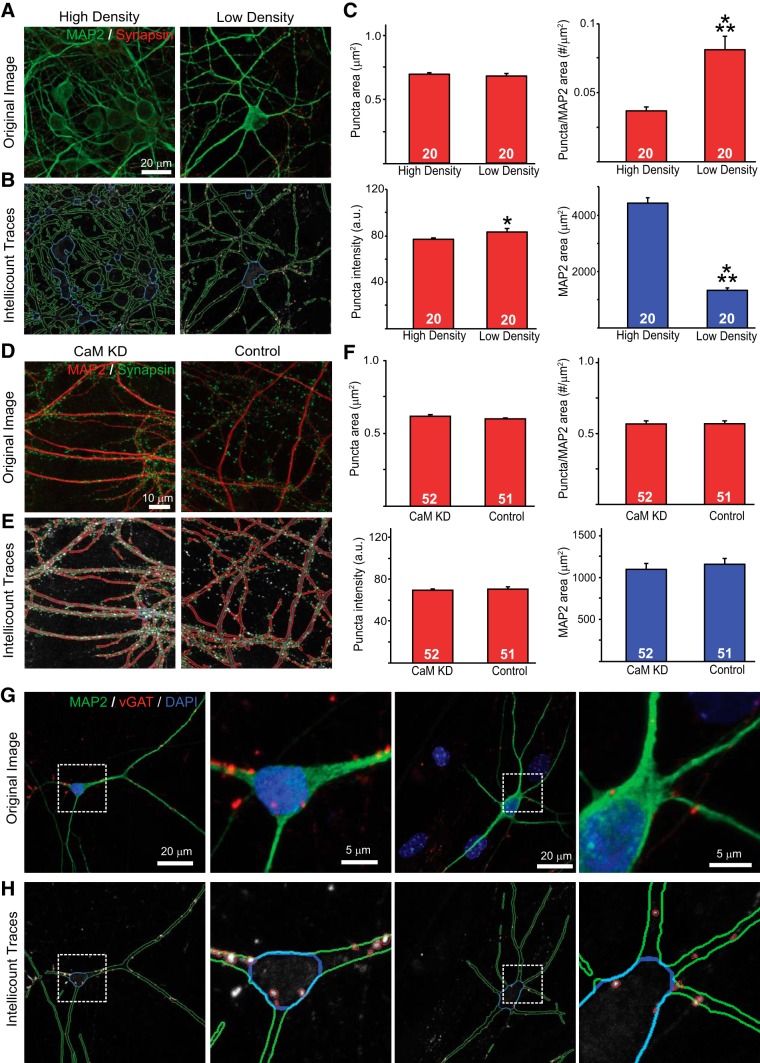
Validation of *Intellicount* under different culture and staining conditions. ***A***, Representative input images of high (left panel) and low (right panel) neuronal densities. ***B***, Corresponding traces provided by *Intellicount* demonstrating differences in segmented MAP2-positive areas. ***C***, Quantification of puncta number and properties (red graphs) provided by *Intellicount* under high and low neuronal densities. Total segmented MAP2-positive area shown in blue graph; *n* = 20. ***D***, Representative fields for lentiviral-infected CaM knock-down (CaM KD) and control (empty viral vector) expressing hippocampal neurons and (***E***) traces provided by *Intellicount*. Here, MAP2 “show correlation” is selected, which highlights MAP2 signal at 50% intensity level. ***F***, Quantification as in ***C*** displaying no differences in synapse formation as described previously (Pang et al., 2010); *n* = 52 and 51 for CaM KD and control, respectively. ***G***, vGAT staining (red) and MAP2 staining (green) in inhibitory human iNs. ***H***, *Intellicount*’s trace of vGAT puncta and MAP2. A total of 60 images were analyzed. White boxes show cropped areas for visualization. Numbers in bars indicate the number of images processed from that condition. Data are displayed as mean ± SEM. Statistical tests were performed using two-tailed Student’s *t* test, where *p < 0.05 and ***p < 0.001.

To further demonstrate the robustness and reliability of the program to quantify synapses in images from different treatment conditions, we tested the program over three alternative conditions: (1) high versus low neuronal densities, (2) IF imaging data collected by another research group with a defined molecular manipulation, and (3) images obtained using a different primary antibody, anti-vGAT with an alternative anti-MAP2 antibody. Fields with high and low neuronal densities were imaged from the same coverslip derived from mouse hippocampal cultures at DIV 8 of our synaptogenesis time course. High cell density images were considered dense if there were more than five cell bodies and MAP2-positive structures in the images. Low density images were considered low if there were only one or two cell bodies present and limited MAP2-positive area. *Intellicount* was able to detect puncta in both images (Fig. 4*A*,*B*), showing comparable puncta area (Fig. 4*C*). The program did, however, detect a slight difference in intensities between the groups. Interestingly, while the program did detect the increase in MAP2-positive area with the high-density images, the synapse density was inversely correlated with MAP2-positive area (Fig. 4*C*), which is consistent with a previous report (Cullen et al., 2010).

To further validate the program, we obtained IF images from a previous report (Pang et al., 2010) to be processed in *Intellicount*. The original report demonstrated that there was no significant impact on synaptic properties after Calmodulin (CaM) knock-down compared with control. To validate the program against this finding, we obtained ∼50 images from both conditions, which had been stained for MAP2 and synapsin (Fig. 4*D*). *Intellicount* was able to accurately identify synaptic puncta within these images as well as MAP2 correlations (Fig. 4*E*). The program did not detect any significant changes between all three synaptic parameters, as well as MAP2 area between control and CaM knock-down conditions (Fig. 4*F*), consistent with the finding this report. The reproduction of this data serves as further validation for *Intellicount* and demonstrates its ability to reliably identify synaptic structures under different experimental conditions and culture types.

Lastly, we sought to demonstrate that alternative antibodies against synaptic proteins other than synapsin that could be used for tracing of synaptic puncta using *Intellicount*. We immunostained inhibitory human iNs for vGAT and MAP2 (Fig. 4*G*,*H*). Sixty images were obtained from three different culture samples. Similar to synapsin quantification, vGAT IF puncta could be identified with apparent accuracy, quantified, and normalized to MAP2 signal (Fig. 4*G*,*H*). The average area over the 60 images was 0.57 ± 0.11 μm^2^, and the intensity was 72.7 ± 13.2 a.u.

Taken together, these data demonstrate the robustness of *Intellicount* to handle different sets of images collected, and that *Intellicount* can be used for a reliable quantification from different culture types and experimental conditions.

### *Intellicount* automates post-processing analysis and data representation

**Figure 5.**
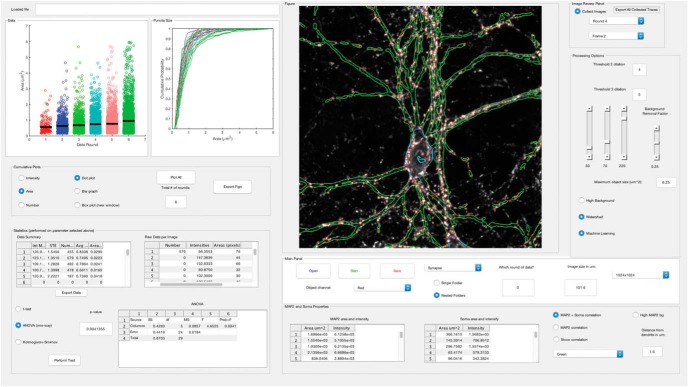
*Intellicount*’s GUI. Screenshot of the *Intellicount* analysis platform displaying results for synapse size from the synapse formation time course (data panel, upper left panels) depicted as dot and cumulative plots and an example analyzed field (figure panel, upper middle pane). For details on operation, please refer to the user guide accompanying the software.

To facilitate quantitative analysis of large datasets such as those demonstrated, we designed *Intellicount* to feature a user-friendly GUI, with automated figure generation and statistical output (Fig. 5). Raw data can be exported directly to Microsoft Excel, which includes folder name, file name, and all averaged properties for each image, including MAP2 and soma characteristics. The “save” button additionally allows the user to collect all raw data in a single, four-dimensional matrix retaining raw data of individual puncta associated with each image. Traces of images can be collected and exported using the “collect images” function, for display (Fig. 3*A*, bottom row).

**Figure 6.**
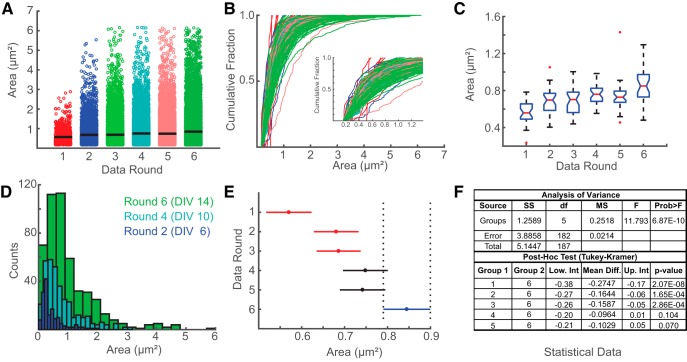
*Intellicount* provides a multifunctional analysis platform with commonly used graphical data representation and statistical methods. ***A-C***, In addition to bar graphs, data distribution can be directly visualized and exported as (***A***) dot, (***B***) cumulative fraction (for puncta area and intensity), or (***C***) box-and-whisker plots. Note, rounds here are reported according to folder nesting order and correspond to increasing time points in the synapse formation analysis presented in bar graph form previously (Fig. 3). ***D***, Histograms for areas of identified puncta are collected for each image for three increasing time points. Here, one image taken from three different rounds are overlaid to demonstrate the shift toward larger puncta during maturation. ***E***, Statistical comparisons can be directly performed in *Intellicount*’s analysis platform allowing visualization of significantly different groups. Rounds highlighted in red are significantly different from the round selected by the user in blue. ***F***, Computation of statistical test data including *p* values for one-way ANOVA and *post hoc* analysis (Tukey-Kramer). Note, box notches (***C***) and line lengths (***E***) define the boundary of 95% confidence intervals for the data and the mean for each round, respectively. Thus, in ***E***, rounds colored with red (rounds 1–3) are statistically different (at *p* < 0.05) from the round selected in blue (round 6), while rounds colored in black (rounds 4–5) are not.

We designed *Intellicount* to provide a single user-friendly platform for immediate statistical analysis and graphical representation. Our program provides to the user three options for plotting data for each parameter measured (intensity, area, and number). The parameter and the output mode can be selected in the GUI before plotting. It can also display data as a bar graph, dot plot, or box plot, as well as depict the mean and 95% confidence intervals of individual rounds for visual analysis of potential group differences (Fig. 6). To highlight these plotting features, we used the area data obtained from the synaptogenesis time course in cultured mouse hippocampal neurons. The dot plot displays all individual results, showing the overall trend toward a corresponding increase in area over time (Fig. 6*A*). Data round, which refers to the order of nested folders, is the default *x*-axis label. In this case, round 1 is DIV 4, round 2 is DIV 6, etc. Additionally, the data can be plotted as a cumulative fraction curve (Fig. 6*B*) or as a box plot (Fig. 6*C*). Boxplots are generated using MATLAB’s boxplot command, where the red line indicates the median, the box edges are first and third quartiles. The dashed line edges, “whiskers,” show the range of nonoutliers. Outliers are plotted as single points in red. For individual images, a histogram of puncta area is automatically generated during analysis. Overlaying histograms for a single image from rounds 2, 4, and 6, shows again the trend toward a greater number and proportion of larger puncta (Fig. 6*D*). Taken together, these features allow users to visually compare how puncta are distributed between rounds and within images. Additionally, when ANOVA is selected as the statistical test, the ANOVA table will display in the GUI. *Intellicount* also automatically runs the multcompare command, which performs a *post hoc* Tukey-Kramer test for multiple comparisons between individual groups. This command generates a new window with a plot that allows the user to select individual groups (Fig. 6*E*, blue line), displaying which groups have significant differences (Fig. 6*E*, red lines). Lastly, it will provide a table displaying the 95% confidence interval of the mean differences between groups and associated *p* values between groups (Fig. 6*F*), enabling the user to evaluate the level of statistical significance for all possible pairwise comparisons.

## Discussion

Validated, high-throughput techniques allow researchers to generate, test, and evaluate hypotheses more rapidly and thus greatly advance scientific discovery. They also provide an unbiased, standardized platform for analyzing datasets and hence facilitate scientific rigor and data reproducibility. User-friendly bioimaging processing software tailored for specific platforms is in great need (Carpenter et al., 2012) and quantification of synaptic protein puncta visualized by IF (or a fluorescent tag) remains a widely used technique in neuroscience that is often cumbersome and prone to bias and human error. Semiautomated methods speed up this process, although there can sometimes be a trade-off between accuracy in tracing signals and processing speed. Importantly, thresholding methods may artificially reduce puncta size, capture background signal as ROIs, or miss low-intensity puncta containing pixels beneath the threshold. To address these drawbacks, we developed *Intellicount* as an analysis paradigm and software program, which performs synaptic protein analysis of IF puncta via ML on a series of images of any size, providing data on the puncta number, mean area and fluorescence intensity. As we demonstrate, this platform provides an efficient and largely unbiased analysis of synaptic IF puncta that can be used for analyzing synapse formation across a wide range of puncta densities and properties under different experimental conditions.

### ML for improved puncta identification

We developed *Intellicount* to employ an unsupervised structure-guided algorithm (i.e., guided by mathematical optimization equations rather than an empirical teaching approach), which corrects traces based on IF image gradients (Fig. 1). Gradient magnitudes, which *Intellicount* uses as a structural template, are generated through application of a Sobel gradient operator. This approach focuses the puncta trace toward the periphery of the structure, immediately before an upward slope in signal occurs. Puncta size cutoffs and MAP2 correlation are applied before ML optimization, so that only the puncta of interest undergo ML optimization. By conjoining puncta identification with the MAP2 correlation, puncta over a wide range of IF intensities can be traced within a short distance of dendrites or neuronal somata.

Also demonstrated, *Intellicount* provides a rapid, multi-threshold analysis which is at least comparable to thresholding approaches in Fiji, however, providing improvements on puncta segmentation and background pixel inclusion (Fig. 2). When compared to a manual trace, *Intellicount* was not able to recapture the full area. While manual traces capture all (even very low-intensity) pixels in the ROI tracing, *Intellicount* optimizes the trace according to the outermost second gradient, which may not include those very low-intensity pixels. Furthermore, since the mean overlap between the trace of a puncta and the gradient is used to determine optimization, traces are most optimized for the average-shaped puncta in the image. Therefore, oddly shaped puncta, which can be traced with precision manually, may not be precisely traced using this approach. Nevertheless, the overall benefit of the ML and image segmentation paradigm is evident by its ability to discriminate differences between experimental conditions efficiently and reliably without image cropping or carefully set thresholds.

### *Intellicount* performs a robust analysis of different datasets

We also demonstrated that *Intellicount* is capable of handling a large dataset of different experimental conditions (time points, molecular manipulations, culture densities and antibodies). Using synaptogenesis time course experiments (Fig. 3) in cultured hippocampal neurons, we demonstrate that *Intellicount* can recapitulate relative differences between time points as shown in previous reports using other image-processing methods (Mozhayeva et al., 2002; Chanda et al., 2017). Here, increases in both fluorescence intensity and puncta area are consistent with increases in synaptic maturation over time as can be observed by electron microscopy as increases in the length of synaptic contact zones and number of vesicles per synapses (Ichikawa et al., 1993). *Intellicount*’s ability to detect this increase demonstrates its utility for quantifying relative differences between conditions over a range of puncta densities and intrinsic puncta characteristics. The program captures low-intensity puncta from earlier time points as well as high intensity puncta of later time points, which is likely due to the use of multiple thresholds. Furthermore, *Intellicount* is able to recover most of the lost area through its ML optimization, which would likely be lost simply through default threshold-based segmentation alone (Figs. 1, 2). However, our program reaches a limit in its ability to segment puncta when the signal becomes saturated, a limitation of any imaging software that bases its processing on signal intensities. If closely located puncta have saturated pixels with little intensity distinction between them, *Intellicount* will probably see these puncta as one. This is likely a caveat of any automated synaptic analysis program, and can be improved or eliminated through careful imaging technique and acquisition methods that do not result in significant signal saturation.

We further validated the program under different experimental conditions and reagents (Fig. 4). *Intellicount* is able to comparably identify puncta from images containing regions of high and low neuronal densities derived from the same specimen (Fig. 4), suggesting it is not substantially impacted or biased by cell density in ROI identification. In this analysis, we did observe a significant increase in the number of MAP2-correlated puncta in the low density condition, consistent with a previous report suggesting an inverse relationship between synapse density and neuronal number (Cullen et al., 2010). Furthermore, this experiment demonstrates that *Intellicount*’s MAP2 segmentation function is capable of reporting differences in neuronal densities which can be a useful additional comparator for analyses (Fig. 4*C*, lower right graph). To demonstrate that *Intellicount* can reproduce existing datasets and detect nondifferences between conditions, as well as handle data obtained by other methods, we processed images from Pang et al. (2010) in an automated format. Importantly, *Intellicount* was able to reproduce these data demonstrating that CaM knock-down does not significantly alter synapse formation in hippocampal neurons cultured at similar cell densities (Fig. 4*D-F*). Finally, to test the validity of *Intellicount* in tracing IF puncta visualized by a different antibody against a different synaptic protein, we performed vGAT staining on inhibitory induced neuronal cultures (Yang et al., 2017) and observed punctate signals correlated to neuronal structures.

Collectively, these analyses demonstrate that the program is capable of running a variety of image sets obtained with varied neuronal densities, image acquisition methods, and primary antibodies for puncta analysis, with little adjustment in processing settings. Importantly, these results, just as those obtained from our synaptogenesis time course experiment, were obtained from full images without cropping images to capture specific locations or subfields and thus removing potential biases from the analysis.

### GUI aids usability for data analysis and presentation

IF staining for synaptic markers often includes some diffuse signal in the cell nucleus or proximal neuronal processes, which can cause aberrant ROI identification. Therefore, we have also included a background removal factor, which discriminates ROIs using a gradient intensity image. True puncta signals should have a significant rise over background and a quick decay, which produces a high gradient intensity, which can be used to filter nonspecific ROIs. Application of this background removal factor is most important when considering more saturated images and analyses where the somata are included. If it is set too low, it may include smaller background ROIs in the analysis, reducing the average puncta size. If set too high, the program will remove true puncta from the analysis. We therefore recommend that all users optimize this parameter before running datasets.

The “plot all” option allows the user to visualize the data of the experiment in three ways for three puncta measurements (intensity, area, and number). These figures can be exported and saved as a variety of image types to assist in figure preparation. For data export, we have built in a button (“export data”) which allows the user to capture data from all rounds and images. The data are compiled into a Microsoft Excel sheet, which is saved with a time stamp into the original directory, where the .m-file (MATLAB file) is located. This enables the user to capture data from a run for additional/alternative statistical analyses or other analysis outside of the GUI. Graphs produced in the GUI can also be exported. Overall, the GUI is designed to be as inclusive as possible, allowing a user to upload a full dataset of either 8- or 16-bit RGB images and conduct a statistical analysis on that dataset, all within a short time period.

We sought to provide the user with an easy-to-use interface (Fig. 5) featuring common graphing options to compare groups, as well as built-in statistics for testing differences. Significantly, running individual images or individual folders adds time to processing a series of images, even in semiautomated programs. To maximize the efficiency of data processing, we implemented a nested folder input paradigm, which allows the user to upload a folder containing grouped subfolders of all images to be analyzed. *Intellicount* can be used for most common image formats (e.g., TIFF, JPEG). Resolution and image size (*x* and *y* dimensions in micrometers) must be set directly in the GUI before running the program. Importantly, there are no restrictions on field size or shape, which allows precropped images (from the same original dimensions and resolution) to be run together simultaneously, even if the cropped dimensions differ between images. For display of these cropped images in the GUI, *Intellicount* zero-pads the smaller images until they reach the largest column and row sizes of any given image. Therefore, a user can prepare a dataset where each folder contains a different condition for analysis, and upload the entire experiment into the GUI at once, rather than sets of images individually. After the run is completed, the user can investigate individual images from each round in the GUI or export them by organized round into folders to evaluate the accuracy of traces of the identified puncta and MAP2.

*Intellicount* offers three statistical tests: Student’s *t* test, ANOVA, and the Kolmogorov-Smirnov (K-S) test for normality. Selecting ANOVA will also provide the user with automatic *post hoc* comparison between groups using the Tukey-Kramer’s *post hoc* test (Fig. 6*E*). The K-S test can currently only be applied to single image area data. This option offers the opportunity to see whether the identified areas provide a sample of puncta from a normal distribution, as required for *t* tests or ANOVA analyses.

Based on the design strategy, *Intellicount* has some limitations. While ML greatly improves identification of most IF puncta, not every punctum may be perfectly traced. The optimization is focused around the average-sized, rounded puncta and functions best on the brightest puncta where gradients are clearly defined. Secondly, since *Intellicount* utilizes thresholding as its primary means of segmentation, bright IF signals with low background are recommended for optimal processing, as is likely true for any IF image analysis program. Also, while the background removal factor can drastically improve high-background image results, there may be a trade-off between puncta captured at the low-intensity end if the background correction is set too high, and capturing some background pixels as ROIs if set too low. The user must determine how strict to set this cutoff for ROI identification, and some optimization may be required before running an image set. Also, we demonstrated that *Intellicount* is also able to segment dendrites and somata for correlation with identified synaptic puncta. Segmentation of dendrites and soma are performed based on the correlation channel staining. While *Intellicount* often successfully identifies somata in low density cultures, it may not as accurately segment them from high-density cultures (Fig. 4*A*,*B*), since closely-located dendrites may appear to the program as a distinct cell body. Inaccurate segmentation of somata may also occur when a MAP2 stain is used, as the intensity is often lower in the cell body than the dendrites. Thus, soma segmentation may not be optimal under all conditions (for example high-density cultures; Fig. 4*B*) and can be toggled on or off according to user preference. It is also important to note that the reported value for soma area is only the largest soma segmented from the image (see additional comments in the user guide; Extended Data 1). This is to reduce the impact of smaller, nonspecific objects biasing the soma area results, which can be erroneously segmented from the MAP2 channel. Lastly, because the ML algorithm uses a built-in watershed segmentation, it can be subject to the plateau problem (Nikodem, 2009), resulting in the possible inflation of puncta number. If puncta tracing and number appear artificially inflated, this can be improved by toggling the “watershed” option off, but at the risk of not achieving full separation between closely-located puncta.

*Intellicount* was designed to be a user-friendly, fully-automated program to perform synapse quantification and analysis. Built-in graphical and statistics features based off the MATLAB Image Processing and Statistics toolboxes offer a range of features for the user to automate data analysis without transferring raw data to a separate program. The nested folder option also greatly improves processing of large amounts of data. All these options taken together provide a robust program for high-throughput quantification of synaptic puncta.

### Future directions

While *Intellicount* is already fully-automated in its current capabilities with different imaging formats and conditions, we plan to expand its function to include colocalization and high-content imaging analyses. Furthermore, since *Intellicount* is primarily designed for quantification of puncta, only puncta number, area, and intensity can be displayed and analyzed in the user interface. We plan to improve soma segmentation and incorporate analysis within the GUI. Moreover, *Intellicount* features limited but commonly used statistical and graphical functions which can likely be extended in future versions. Finally, while *Intellicount* is optimized for identifying synaptic protein puncta in neurons imaged by laser scanning confocal microscopy, we would like to extend its versatility to be scalable to other imaging modalities (e.g., super-resolution microscopy) and applications such as cell counts and sub-cellular organelles, which will likely require further refinements in the ML design. Moreover, since we provide the research community *Intellicount* in an open source format, it can be used as a foundation for further improvement by the community.

## Conclusions

We have developed and tested a novel program, *Intellicount*, which automates synaptic puncta analysis. ML, which is built into the program, improves puncta tracing and minimizes operator error or bias. Furthermore, a nested folder analysis option and built-in statistics and graphical representation provide the user with a robust experimental analysis of puncta properties. All these features are contained within a single open-source GUI found online at http://license.rutgers.edu/technologies/2018-013_intellicount along with a simple user guide and thus offer a powerful platform for analysis of synaptic properties which will hopefully aid in the dissection of synapse formation under normal and disordered conditions.

## Synthesis

Reviewing Editor: Menno Witter, Norwegian University of Science and Technology Decisions are customarily a result of the Reviewing Editor and the peer reviewers coming together and discussing their recommendations until a consensus is reached. When revisions are invited, a fact-based synthesis statement explaining their decision and outlining what is needed to prepare a revision will be listed below. The following reviewer(s) agreed to reveal their identity: Alexander Chubykin, Konark Mukherjee

The manuscript is significantly improved: the authors have now looked at 6 different developmental time points representing different culture density. These experiments have both validated the algorithm and also provided meaningful information about the developmental profile of these cultures, which is consistent with the previously published literature. Furthermore, the authors have also tested different antibodies.

The authors have also now detected and corrected (following our reviews) the mistakes in the error bar calculations.

The authors have now added and described the export data/analysis function for further posthoc analysis.
