# Supplementary material for: Intellicount: High-Throughput Quantification of Fluorescent Synaptic Protein Puncta by Machine Learning
Source: eNeuro. 2017 Dec 6;4(6):ENEURO.0219-17.2017. doi: 10.1523/ENEURO.0219-17.2017 (PMC5718246; doi:10.1523/ENEURO.0219-17.2017)
Supplement: Extended Data 1 — File contains two MATLAB files and user guide. Download Extended Data 1, ZIP file. [file sup_enu-eN-MNT-0219-17-s02.zip › Code on website/Intellicount User Guide.pdf]

Intellicount User Guide

This guide is designed to serve as a quick start guide for new users. Individual panel screenshots are explained in detail below. The panels are displayed below in the order of use. This guide is divided into two sections: Pre-processing (blue background with blue circles) and Post-processing (green background with green circles). Pre-processing includes the Main Panel, MAP2 and Soma Properties Panel, and the Image Review and Processing Options Panel, which assist in experimental setup and running conditions. Post-processing includes the Cumulative Plots Panel and Statistics Panel, and are performed after processing.

PRE-PROCESSING

Main Panel - Processing basics:

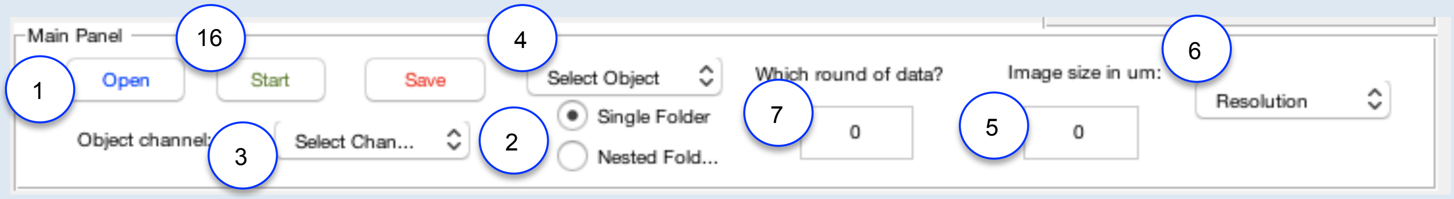

1. Click Open, and select the folder which contains the images you would like to run. If you are running a nested folder option, select the folder (nested folder) containing the folders (sub-folders) which contain the images. For example:
    a. Single Folder
        i. Folder 1 **(Select this level)**
            1. Image 1
            2. Image 2
            3. Image 3
    b. Nested Folder
        i. Nested folders **(Select this level)**
            1. Sub-folder 1
                a. Image 1
                b. Image 2
                c. Image 3
            2. Sub-folder 2
                a. Image 1
                b. Image 2
                c. Image 3
2. Choose the Folder options that correspond to your folder setup (either Single or Nested).
3. Choose the object channel. E.g., if your puncta are in the 488 channel, select “Green”. Only “Red”, “Green” and “Blue” options available. (Default set to “Red”).
4. Choose object type (either “synapse” or “cell”) under “Select Object” dropdown menu. (Default is “synapse”).
5. Enter the image size in μm. (Default: 101.9 μm). **Note:** if the image is cropped, choose the size of the original image. Taken together with the resolution, this will determine pixel size for puncta areas.
6. Select the image resolution. (Default: 1024 x 1024). If other is selected, the program will use the width of the input image for size.
7. Enter the Round number. Here, Round means a single dataset, e.g., control, or experimental condition #1. If you would like to position data in a certain order, you may alter the order with this feature by numbering the rounds differently. If left empty (or 0), Round defaults to 1. Under the “Nested Folders” condition, Round updates automatically each time a new sub-folder is encountered.

MAP2 and Soma Properties Panel - If performing a MAP2 correlation:

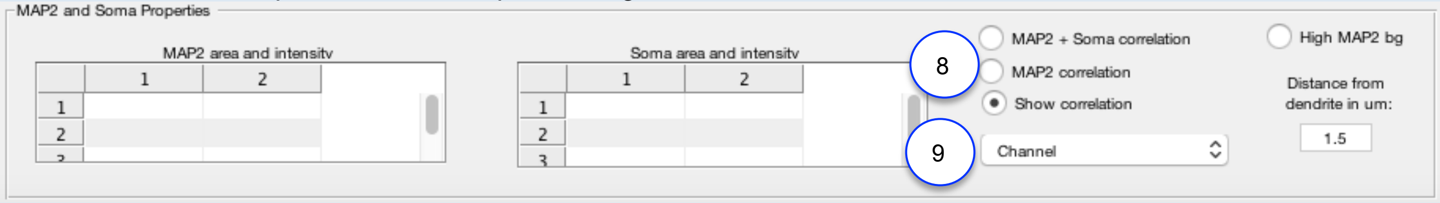

8. Select MAP2 options.
    a. "MAP2 + Soma correlation" captures all puncta within a defined distance (see "Distance from dendrite in um", default 1.5) of MAP2 in dendrites *and* all puncta within identified somata.
    b. "MAP2 correlation" only captures puncta within a defined distance from the dendrite.
    c. "Show correlation" is for visualization only. If you would like to see the MAP2 channel overlaid as 50% grayscale intensity on the puncta channel, select "Show correlation". Otherwise, remove selection.
9. Choose the MAP2 channel. Options are "Red", "Green" or "Blue". (Default: "Blue"). **Note**: whichever channel is chose for MAP2, all dendrite traces will show in this color. The soma will be traced in whichever color has not yet been used. E.g., if puncta are red and MAP2 is green, the soma will be traced in blue.

Additional notes on MAP2 correlation: 1) if MAP2 channel has a high background, select "High MAP2 bg". This will shift the threshold used for MAP2 segmentation and may improve results. 2) The distance from dendrites can be altered based on the puncta studied. The default is 1.5 μm. This includes all puncta within 1.5 μm of a dendrite on either side.

Image Review and Processing Options Panels (optional):

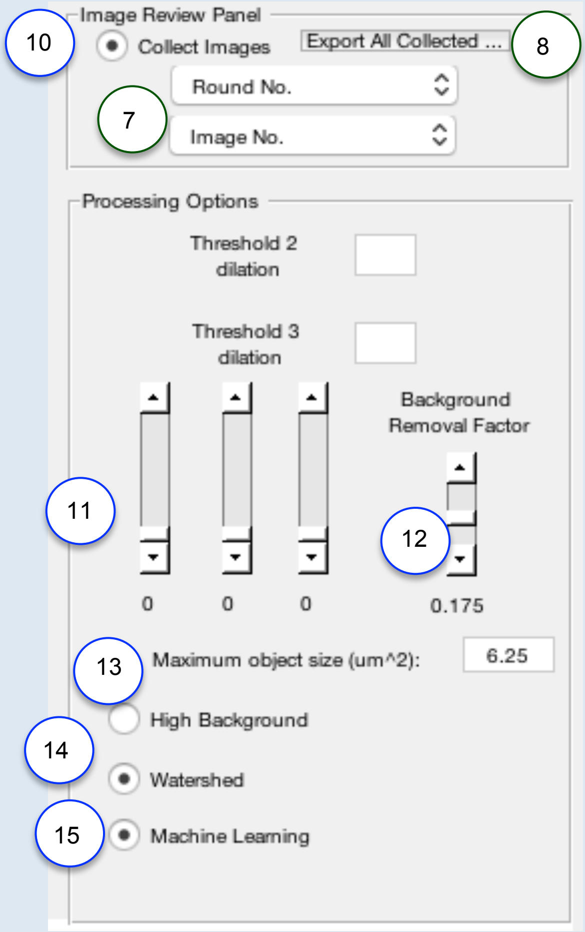

10. "Collect Images" can be toggled off if the current memory space is insufficient to store all traces. This may be the case will large numbers of files. (MATLAB will give an error in the command window if this is the case. Reset the GUI and toggle this off to re-run.)
11. Default thresholds are set to 30, 70, and 180 (as per an 8-bit scale). These may be changed as needed, but the machine learning algorithm should account for discrepancies in non-optimized threshold settings. If other thresholds are desired, they may be changed, but this must be done prior to starting the analysis. **Notes**: 1) if default thresholds are desired, make sure sliders read "0" prior to start. 2) For 16-bit images, threshold values are converted to a 0-255 scale before application, and no change needs to be make to accommodate these images.
12. The "Background Removal Factor" thresholds out background signal. This greatly aids in the capture of true puncta located in the soma (or other high background areas). Lowering the value will capture more objects in the field. Raising will capture fewer. (Default is 0.175). For high backgrounds, we recommend 0.25.
13. Maximum object size (in μm^2) can be adjusted if needed. Default: 6.25 μm^2. **Note:** due to rounding, the true value may be slightly different.
14. If puncta channel has many saturated puncta and a high level of background, the analysis may be aided by selecting "High Background". This raises the default thresholds to 85, 120, and 230. It also removes a sharping filter in the image pre-processing.
15. Machine learning and Watershed may be toggled on and off. It is recommended that both remain on for optimal analysis. If over-segmentation occurs, that is, too many watershed lines are drawn, the Watershed option may be removed.
16. Click Start on Main Panel to begin analysis.

POST-PROCESSING

Cumulative Plots Panel:

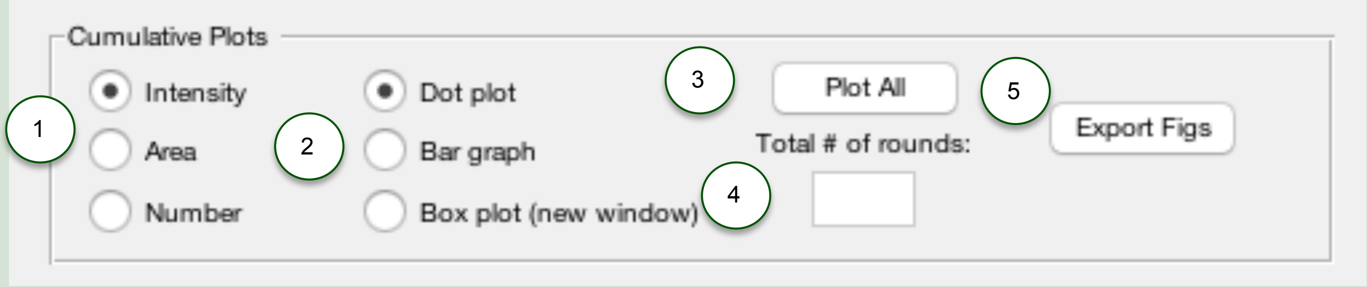

1. Once the analysis is completed, the parameters may be selected for plotting. Select the parameter of interest.
2. Next, select the graphical style. After step 3, if Box plot is selected, MATLAB will provide the plot in a new window.
3. Select Plot All to perform the desired plotting action.
4. Total # of rounds will display the total number of rounds in the analysis. This is automatically populated if Nested Folders were run. If single folders were run as successive rounds, you must update the total number of rounds analyzed in order to plot all rounds.
5. Export Figs allows you produce the plotted figures as separate windows for saving.

Statistics Panel:

Important note: Statistics can only be performed on one parameter at a time. The parameter to be analyzed must first be plotted in the Cumulative Plots Panel above. Doing so provides the GUI with the data needed to perform the statistical analysis.

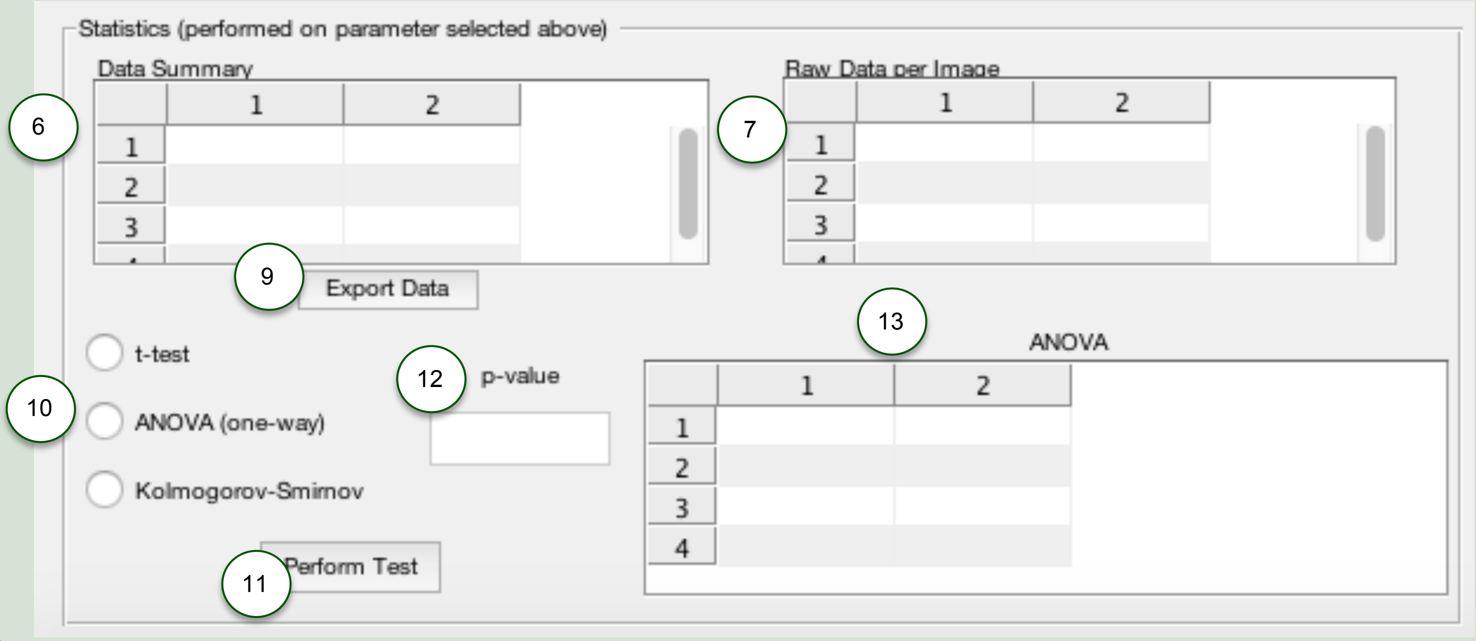

Statistics Panel continued

6. Data Summary provides the averaged data from each image. Images will be listed by row. Data are exported to this table in the following order: intensity mean (Int Mean), intensity standard error (STE), puncta number (Number), average area (Avg Area (um^2), area standard error (Area STE).
7. Raw Data per Image will display the raw data of a selected image. The data are displayed in the table as Number, Intensities, and Areas (pixels). **Note:** for both 6 and 7, the tables can be updated by selecting new rounds and new images from the Image Review Panel. For the data to be updated, both a **Round AND an Image** must be selected. (See Image Review Panel above for location.)
8. Images may also be exported from the Image Review Panel. Selecting Export All Collected Images will create in the open directory folders according to the round numbers of all images with their traces.
9. Export Data allows you to obtain a copy of all summary data from the analysis. The file name will be of the template: Data SummaryMM-DD-YYYY H:MM AM/PM. The data are displayed in columns with the headers: Round, Directory, Image_names, Images, Number, Intensity_avg Intensity_STE, Area_avg, Area_STE, MAP2_Area, MAP2_Intensity_avg, Soma_area, Soma_Intensity_avg, Combined_Area. The file will be located in the same folder as the MATLAB file (.m-file).
10. Select the test you want to run. After step 11, Kolmogorov-Smirnov test will bring up a separate window allowing you to select the image whose areas you want to test for normality. Selecting ANOVA will automatically perform a post-hoc Tukey-Kramer test. Two new windows, one table and one graph will appear. The table displays p-values between individual groups. The graph shows the 95% confidence intervals between the means of groups and allows you to select one group to compare between the others.
11. Select Perform Test to run the desired test.
12. The p-value will be displayed here for both t-test and ANOVA.
13. The ANOVA table will populate here.

Additional comments:

1. When MATLAB encounters an error, it will display red text in the main command window. If you encounter an error, review your selections for the run and make sure everything is correct. To re-try the program, change the directory in MATLAB to the directory with the .m-file and select Start. Otherwise the MATLAB will not locate the program.
2. When multiple analyses are run, the GUI may have plotting errors (plotting multiple datasets at once). To correct this error, close the GUI and re-open it. This resets all stored values in the GUI.
3. For segmented somata, only the largest soma is reported for area. The mean intensity for all somata found in the image is reported for intensity.

© 2017 Rutgers, The State University of New Jersey, All rights reserved. Do not copy or reproduce without permission.
